# Home-Based Exercise in Elderly Patients with Claudication and Chronic Kidney Disease Is Associated with Lower Progressive Renal Function Worsening: A 5-Year Retrospective Study

**DOI:** 10.3390/metabo13010056

**Published:** 2022-12-30

**Authors:** Giovanni Piva, Anna Crepaldi, Nicola Lamberti, Lorenzo Caruso, Natascia Rinaldo, Roberto Manfredini, Pablo Jesus López-Soto, Vincenzo Gasbarro, Fabio Manfredini, Alda Storari

**Affiliations:** 1 PhD Program in Environmental Sustainability and Wellbeing, Department of Humanities, University of Ferrara, 44121 Ferrara, Italy; 2 Department of Nursing, Instituto Maimónides de Investigación Biomédica de Córdoba, 14004 Cordoba, Spain; 3 Unit of Nephrology, University Hospital of Ferrara, 44124 Ferrara, Italy; 4 Department of Neuroscience and Rehabilitation, University of Ferrara, 44121 Ferrara, Italy; 5 Department of Environmental and Prevention Sciences, University of Ferrara, 44121 Ferrara, Italy; 6 Department of Medical Sciences, University of Ferrara, 44121 Ferrara, Italy; 7 Department of Nursing, Pharmacology and Physiotherapy, Universidad de Córdoba, 14004 Cordoba, Spain; 8 Department of Nursing, Hospital Universitario Reina Sofía de Córdoba, 14004 Cordoba, Spain; 9 Unit of Vascular and Endovascular Surgery, University Hospital of Ferrara, 44124 Ferrara, Italy; 10 Unit of Rehabilitation Medicine, University Hospital of Ferrara, 44124 Ferrara, Italy

**Keywords:** peripheral artery disease, chronic kidney disease, exercise, rehabilitation, quality of life, creatinine, kidney function, hospitalizations

## Abstract

This observational study aimed to monitor the 5-year trends of kidney function in patients with peripheral artery disease (PAD) and concomitant chronic kidney disease (CKD) enrolled or not enrolled into a rehabilitative exercise program. Sixty-six patients (aged 72 ± 10, males n = 52) at KDOQI stages III-IV and PAD at Rutherford’s stage I-III were included in the study, with a group (Exercise, EX; n = 32) receiving a 6-month structured pain-free home-based walking program and a group (Control, CO; n = 34) receiving walking advice and optimal nephrological care. Outcomes included kidney function measured through serum creatinine (sCr) and clinical outcomes, including the rate of advance of CKD stages and admission to dialysis, revascularizations, and hospitalizations. At baseline, the two groups were comparable for age, nephropathy, medications, comorbidities, and PAD severity. Patients in the EX group safely completed the exercise program. SCr values were slightly increased in EX (baseline: 2.35 ± 0.32; 5-year: 2.71 ± 0.39 mg/dL) and progressively worsened in CO (baseline: 2.30 ± 0.31; 5-year 4.22 ± 0.42 mg/dL), with a significant between-group difference (*p* = 0.002). The control group also showed a higher number of dialysis admissions (5 vs. 0, *p* = 0.025) and advancing CKD stage as well a higher risks for lower limb revascularization (hazard ratio: 2.59; 95%CI: 1.11–6.02; *p* = 0.027) and for all-cause hospitalization (hazard ratio: 1.77; 95%CI: 1.05–2.97; *p* = 0.031). PAD-CKD patients enrolled in a low-moderate intensity home-exercise program showed more favorable long-term trends in kidney function and clinical outcomes than patients with usual care. These preliminary observations need to be confirmed in randomized trials.

## 1. Introduction

Peripheral artery disease (PAD) affects more than 200 million adults around the world, and its prevalence is rapidly increasing [1,2]. The disease, which may significantly impair walking ability, is associated with a high risk of adverse clinical outcomes [1]. In PAD, the risk of worsening kidney function is enhanced by arterial stiffness, with related markers found to be associated with the early development of chronic kidney disease (CKD) [3], region-specific vascular stiffness [4], metabolic factors [5], and contrast-induced nephropathy related to endovascular procedures [6]. On the other hand, the risk of PAD is increased by 1 to 4 times in the population with CKD, with an incidence rate among patients on hemodialysis of approximately 400 per 1000 patient-years [7]. The frequent concomitant presence of PAD and CKD represents a dangerous mix for vascular aging [7,8] due to the combination of classic risk factors with several additional inflammatory, metabolic, endocrinological, and toxic aggressive factors [7,9]. Patients with PAD-CKD are exposed to higher cardiovascular risk [8], to the onset of a more severe stage of PAD, such as chronic limb ischemia [10], and to worse surgical outcomes, such as a higher rate of reinterventions and amputations [11], which are strongly associated with albuminuria [7]. Moreover, in patients undergoing hemodialysis, PAD is associated with impaired physical function and reduced physical activity [12].

As a further critical issue for PAD-CKD patients, the restricted mobility due to vascular disease and the progressive sedentariness associated with CKD severity [13] deprive patients of the protective effects related to physical activity [14,15,16].

Conversely, in PAD patients, exercise under supervision [17,18], even not exclusively, improves functioning with a potential number of benefits [14,15,19]. In patients with CKD, exercise also improves function and quality of life [20] and is recommended by the guidelines [21]. Higher physical activity levels may also contribute to a stable maintenance of kidney function, as observed among older subjects [22] and in people with CKD [23]. Moreover, in CKD, a protective effect (e.g., an increased GFR) [24] may also be derived from exercise [25] in addition to the strict management of risk factors. However, no data on patients with PAD and CKD are available.

More than 10 years ago, a test in-train out (TiTo) program prescribed at the hospital and carried out at home at controlled speed was developed for PAD patients [25,26,27,28], with positive functional, hemodynamic and clinical outcomes [29,30], and it was successfully translated for end-stage renal disease (ESRD) patients on dialysis [31,32,33].

Based on these previous observations and in the absence of data from the literature, we hypothesized that a concomitant protective effect on renal function and clinical outcomes may occur.

The present retrospective study aimed to evaluate whether TiTo exercise added to nephrological care in PAD patients with CKD was associated with a different stability in renal function over a five-year period compared to a cohort of patients receiving usual care.

## 2. Methods

### 2.1. Study Design and Setting

In a single-center observational study, we considered consecutive patients affected by PAD-CKD recruited from the Unit of Nephrology or Unit of Vascular Surgery at the University of Ferrara Hospital from 2013 to 2015 and then referred to the exercise program at the Unit of Rehabilitation Medicine. The study was approved by the Local Ethics Committee (CE-AVEC) (approval number 277/2019), but due to the retrospective nature of the study, written informed consent was not attainable from all participants. The STROBE guidelines were followed to report the study.

### 2.2. Subjects

Patients were included in this study if they had PAD at the stage of claudication (Rutherford’s category 2–4) with concomitant CKD at KDOQI stages III or IV. Patients were allowed to take any class of medications (including beta blockers, diuretics, oral antidiabetic drugs, etc.). Patients with severe heart failure (NYHA class IV), absolute contraindications to exercise training (e.g., unstable angina, uncorrected anemia < 10 g/dL), or a known life expectancy below 12 months were excluded from the study. A vascular surgeon diagnosed PAD in each patient by performing an echo-color-doppler instrumental examination to the lower limb arteries (from iliac to tibial arteries, in both limbs) looking for arterial stenoses and/or occlusions as recommended by the guidelines.

Patients enrolled in the exercise (EX) group underwent the Test in-Train out (TiTo) rehabilitation program, while a parallel group of patients with the same inclusion criteria did not, acting as a control group (CO) that followed traditional nephrology and vascular follow-up care.

### 2.3. Exercise Group: Training Program

Patients in this group were previously enrolled in the TiTo home-based pain-free exercise program [25]. This structured low-intensity program included two daily 10-min pain-free walking sessions performed following a 1:1 walk:rest ratio. The training speed, which was converted into a walking cadence (steps/min) and maintained at home by the use of a metronome, was slower than the individual’s walking speed at the beginning (approximately 60%) and progressively increased weekly. A training diary to be compiled daily was provided to each patient, ensuring exercise execution at each follow-up visit at the hospital. The program usually lasted for 6 months, but longer timeframes were possible in cases of temporary interruption (e.g., for intercurrent diseases).

After discharge from the program, patients received recommendations to be physically active and to perform a daily 10-min walking session at a moderate speed at least 4 times per week.

### 2.4. Control Group

Patients in the control group underwent duplex examination without functional assessment. They also received advice to maintain an active lifestyle, according to the guidelines for PAD patients [17], and to follow the optimal nephrological care received by the dedicated specialist. These patients underwent surveillance based on duplex examination and nephrology visits approximately every six to twelve months according to the severity of the vascular and renal diseases.

### 2.5. Study Outcomes

The primary outcome of this study was the variations in kidney function over 5 years in the two groups. Purposely, the overtime variations of serum creatinine (sCr) value and estimated glomerular filtration rate (eGFR) were collected, and the rate of advance to more severe stages of kidney function and of admission to dialysis were noted.

Values reported were obtained from blood samples drawn at least once per year using standard methods after an overnight fast of at least 12 h. Serum creatinine was determined, and the eGFR was estimated using the CKD-EPI creatinine equation. Traditional laboratory parameters were also collected (full blood count, urea, albumin, etc.). The same laboratory at the University Hospital of Ferrara performed the tests using the same instrumentation.

To create a consistent expression of the results, the timepoints considered for the analyses were baseline (year 0, Y0), after 12 ± 1 months (Y1), 24 ± 1 months (Y2), 36 ± 2 months (Y3), 48 ± 2 months (Y4), and the end of follow-up after 60 ± 3 months (Y5).

Secondary outcomes included all-cause hospitalizations and PAD-related revascularizations in the following five years. Outcomes for the EX group were considered after discharge from the training program. Data were censored at the time of death.

Both laboratory and long-term clinical outcomes were obtained between February and May 2022 from the Italian Register of Dialysis and Transplanted Patients (Registro Italiano Dialisi e Trapianti–RegDial), the Regional Register of Progressive Kidney Failure Prevention (Prevenzione Insufficienza Renale Progressiva–PIRP), and clinical records.

### 2.6. Statistical Analysis

Data distribution was verified with a Kolmogorov-Smirnov test. Differences in baseline characteristics for the two groups were evaluated with Chi-squared tests, Student’s *t* tests, or Mann-Whitney tests, as appropriate. A comparison of overtime variations of laboratory biomarkers was performed via repeated measure analysis of variance, considering the group allocation as the between-subject factor. Paired sample *t* tests were also executed to analyze the differences between the single timepoints. Fisher’s test was employed to evaluate the rate of entrance into dialysis.

Kaplan-Meier estimates of the distribution of clinical outcomes (including advance of CKD stage, lower limb revascularization, and mortality) from the time from enrollment to the date of death (or 5-year end of follow-up) with a log-rank test for trends were used to compare the curves of the two groups. Multivariate Cox proportional hazard regression analyses were employed to analyze the effect of several predictor variables on the long-term clinical outcomes for each group. Because of the limited number of events, multivariate HRs were calculated using a forward approach, with an entry limit of *p* < 0.20. Generally, a *p* value < 0.05 was considered statistically significant.

For the primary outcomes, statistical analyses were executed by replacing the missing data (e.g., in case of death) with the method of the last observation carried forward. To test the consistency of the findings, a per-protocol analysis including only patients who presented all the laboratory values for the entire follow-up period was also performed.

All statistical analyses were performed using MedCalc Statistical Software version 20.110 (MedCalc Software bvba, Ostend, Belgium).

## 3. Results

**Figure 1:**
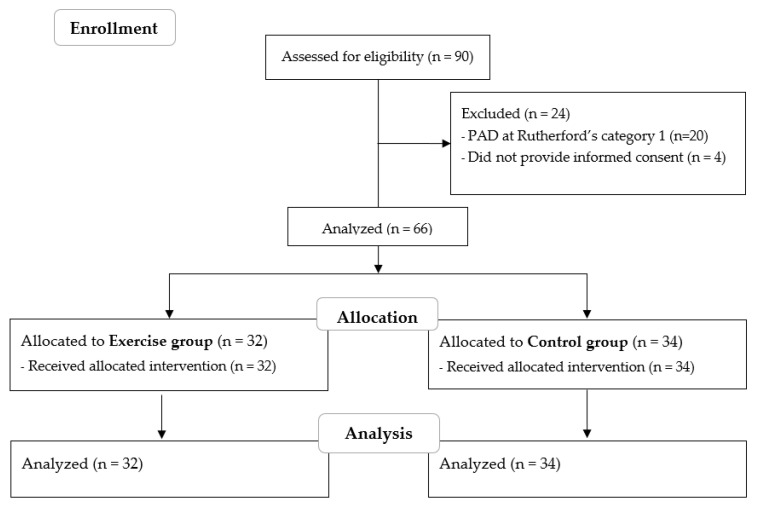
STROBE flow diagram of the study.

A cohort of 90 patients with concomitant CKD and PAD was analyzed for possible inclusion in the study. Twenty-four patients were excluded for noncompliance with the inclusion criteria, and the remaining 66 were analyzed, with 32 belonging to the EX group and 34 belonging to the CO group. Data are reported in Figure 1.

At baseline, the two groups did not present any differences in anthropometrics, comorbidities, baseline nephropathy, use of medications, or PAD severity. Data are reported in Table 1.

All patients in both groups reported optimal adherence to drug therapy. Patients in the EX group safely completed the 6-month program successfully without any adverse events. An average of 5 ± 1 serial visits were performed. For nine of them, a longer duration due to intercurrent diseases was scheduled. The observed adherence to the training was high, with a median of 87% of the walking sessions executed with respect to the prescribed ones. The increase in gait speed during the 6-min walking test was 0.5 ± 0.2 kmh^−1^.

### 3.1. Long-Term Kidney Function

**Figure 2:**
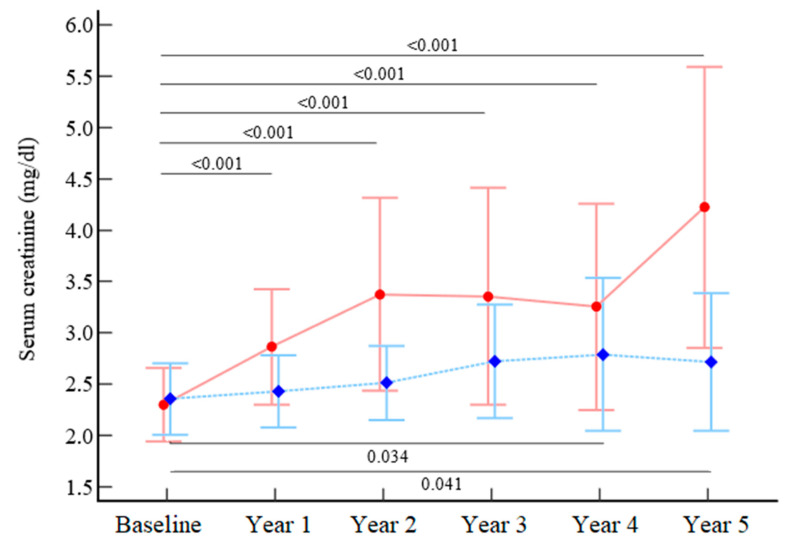
Variations over time in serum creatinine and eGFR in the two groups. Legend: red circle, control; blue diamond, exercise. Data are expressed as the mean and 95% confidence intervals.

Values of serum creatinine in the EX group exhibited a slight increasing trend from Y3 (t = 2.34; *p* = 0.043) with significantly different values at Y4 and Y5 from baseline. In the CO group, a progressively linear increasing trend of sCr was observed (t = 2.80; *p* = 0.012), with significantly different values observed from Y0 to all other time points (from Y1 to Y5). (Table 2, Figure 2). A significant between-group difference was observed in the overtime analysis (*p* = 0.002).

The same trends were observed for eGFR levels, with a significant between-group difference observed in the overtime analysis (*p* = 0.018) (Table 2).

The following per-protocol analyses executed in the subgroup without missing data (EX n = 22; CO n = 19) confirmed the significant difference between groups (*p* < 0.001) in the overtime analysis.

**Figure 3:**
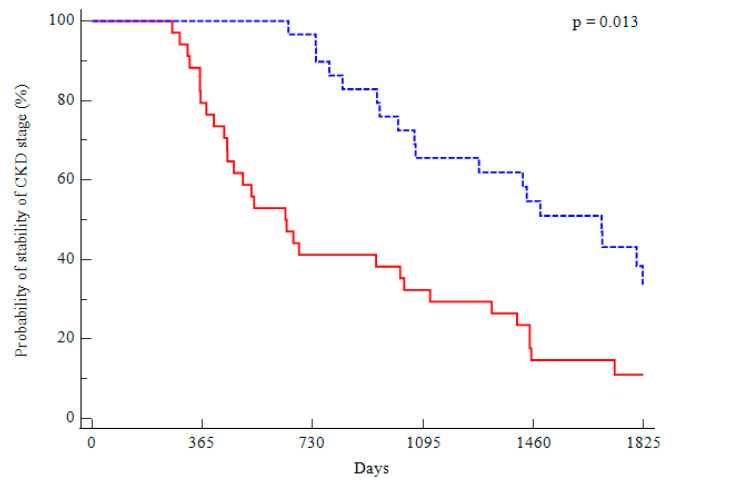
Kaplan-Meier curves showing the probability of changes in CKD stages in the two groups (blue, exercise; red, control).

Variations in CKD stages were also analyzed considering the rate of patients who fell into a more severe CKD stage (*p* = 0.013). A significant between-group difference was observed. Namely, 18 patients in the EX group (56%) versus 30 patients in the CO group (88%) showed a progression of CKD stage, with a significantly higher risk for the CO group (HR: 1.67; 95% CI 1.06–2.63) (Figure 3).

Considering the survivors, at the 5-year follow-up, five patients in the CO group were admitted to dialysis treatment, whereas no patients in the EX group were admitted (*p* = 0.025).

### 3.2. Laboratory Parameters

Values of serum urea showed an increasing trend in the EX group from baseline to the 5-year follow-up (88 ± 34 to 106 ± 40 mg/dL; t = 0.81; *p* = 0.15), whereas a significant increasing trend was noted in the CO group from baseline to the 5-year follow-up (86 ± 38 to 136 ± 71 mg/dL; t = 2.89; *p* = 0.001), with a significant between-group difference (*p* = 0.039).

No between-group differences were observed for the full blood count parameters (Table 2).

### 3.3. Revascularizations and All-Cause Hospitalizations

At the 5-year follow-up, hospitalizations for all causes occurred in 27 out of 32 patients in the EX group and in 33 out of 34 patients in the CO group. The Kaplan-Meier analysis confirmed a significantly higher risk for the CO group than for the EX group (hazard ratio, HR: 1.77; 95% CI: 1.05–2.97; *p* = 0.031).

A total of 64 hospitalizations occurred in the EX group versus a total of 102 in the CO group (*p* < 0.001). The number of hospitalizations per person/year was 0.49 in the EX group versus 0.69 in the CO group (*p* < 0.001).

**Figure 4:**
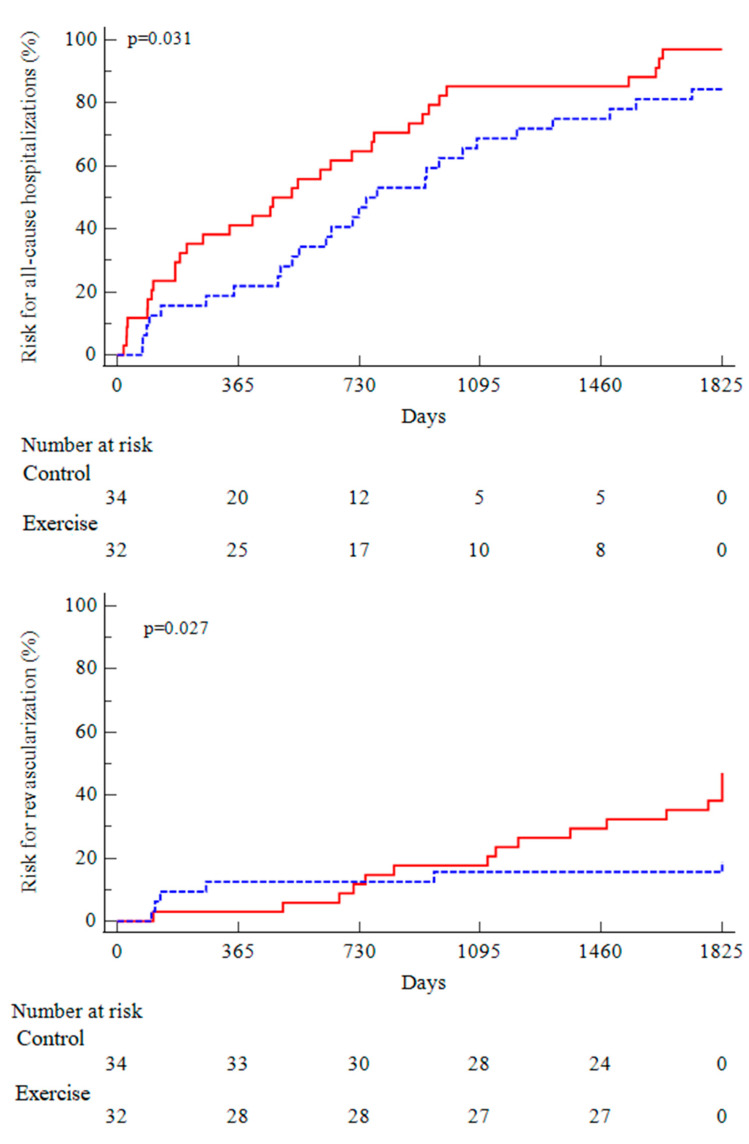
Kaplan-Meier curves of all-cause hospitalizations and peripheral revascularizations in the two groups (blue, exercise; red, control).

Lower limb revascularizations occurred in 7 patients in the EX group and 13 patients in the CO group. The log-rank test also confirmed a higher value for the CO group than for the EX group (hazard ratio: 2.59; 95% CI: 1.11–6.02; *p* = 0.027) (Figure 4).

A total of 25 patients were deceased, 10 belonging to the EX group (31%) and 15 to the CO group (44%), without any between-group difference.

### 3.4. Predictors of Outcomes Probability

Multivariate Cox proportional hazard regression models highlighted that age [HR = 1.04 (95% CI: 1.00–1.07)], baseline serum creatinine levels [HR = 1.72 (95% CI: 1.22–2.44)], and control group [HR = 2.31 (95% CI: 1.26–4.25)] were the only predictors of all-cause hospitalizations in the entire population.

A nonsignificant Cox model was observed for PAD-related revascularizations and admission to dialysis events due to the restricted number of cases. In both models, the only significant protective factor retained was that belonging to the exercise group (HR: 0.24 and HR: 0.32, respectively).

## 4. Discussion

The study showed relatively stable renal function over time in the cohort of PAD patients with CKD initiated to a 6-month exercise program, unlike the usual care population, which showed a progressively greater decline over 5 years and a higher risk of worsening of renal disease stage.

The concomitant presence of PAD and CKD is a major issue due to the high number of vascular aggressive factors, the related unfavorable cardiovascular clinical outcomes, and a possible negative renal disease progression [3,7,9]. This evolution, intermittent and in general unpredictable, may evolve to ESRD, driving the patients to a worsening of quality of life and posing a burden on the health service and the environment [34,35,36]. The traditional control of risk factors and the improvement of the patient’s lifestyle, tackling sedentariness for several protective benefits derived from physical activity [13,15,19], is a recommended action [17,18,35]. Moreover, in the presence of PAD where mobility is restricted and the option of revascularization may increase the risk of progressive renal dysfunction [6,7], rehabilitative exercise may represent the necessary tool. However, to the best of our knowledge, no information in the literature is available on the long-term effects of rehabilitative exercise on kidney function in PAD-CKD patients. We must refer to studies on patients with CKD dealing with the effects of rehabilitation or exercise therapy on renal function after discharge. In particular, few studies have suggested a beneficial effect on kidney function in CKD patients undergoing cardiac rehabilitation [37]. In a retrospective study, patients labeled as active according to attendance (more or less than 1 time/week) showed an improvement in eGFR (+3 mL/min/1.73 m^2^) compared to the nonactive group [38]. In a recent retrospective cohort study in patients at CKD stages III and IV, those participating in the 3-month program showed a mean improvement in eGFR (from 1 to 5 mL/min/1.73 m^2^) with higher benefits for patients with lower baseline values [39]. In addition, studies on CKD patients also showed some benefits of exercise on kidney function [40], and a recent meta-analysis reported a beneficial effect of exercise in non-dialysis CKD patients, with an improvement in eGFR (+2.6 mL/min/1.73 m^2^) with short-term exercise programs (<3 months) [24]. The authors’ conclusion was that exercise therapy may represent a strategy to improve eGFR and targeted risk factors in non-dialysis CKD patients, even in the presence of few studies and a limited number of patients [24]. Another meta-analysis on adult CKD patients without renal replacement therapy observed that exercise training was safe but had uncertain positive effects on proteinuria [41]. On the other hand, a low physical function or a poorly active lifestyle may affect renal function and the rate of decline [35], as observed in older persons [22], in patients after acute myocardial infarction [42] or in those with CKD [23]. Considering the long-term response to exercise, no effects on GFR were observed following a 16-week exercise program in patients with hypertension and CKD but not with PAD. Interestingly, lower ABI together with lower eGFR and higher systolic blood pressure were associated with decreased survival [43]. In the present study, where the population was older, with PAD, and introduced to low-intensity exercise for a longer period, a relatively stable renal function in terms of sCr and eGFR was observed in EX, unlike the usual care group, where a progressive negative evolution occurred after 12 months. The objective result was that, during the 5-year follow-up in the CO group with respect to EX, almost double the number of patients worsened their clinical status, and five patients (15%) went toward ERSD, requiring the execution of over 800 dialysis sessions compared to none of the rehabilitated patients. The study had no elements to explain this clear divergence between the groups, with similar baseline characteristics in terms of nephropathy, comorbidities, and medications. If in the present study a specific role of exercise in the renal outcomes cannot be attributed, the impact of exercise with benefits that may outlast the duration of the TiTo intervention itself [29] and the recovery of mobility in the daily activities may have been a positive synergetic element together with the traditional control of the risk factors.

In addition, the TiTo program, adding 1200–1500 steps per day to the daily routine regardless of external conditions being performed at home [28], represents a significant relative amount of activity for stage IIb PAD patients with restricted mobility and an interruption of their sedentariness [44]. Moreover, the TiTo exercise program, requiring only a few walking minutes of exercise in the absence of pain or fatigue, enables the enrollment of even low-performance subjects, possibly more responsive [39]. A favorable impact on endothelial function was also previously reported following the same exercise program in ESRD patients with changes in stiffness [45] and endothelial progenitor cells in relation to increased mobility [46]. Previous meta-analyses showed that regular exercise had significant beneficial effects on walking ability, blood pressure, heart rate, and BMI reduction in adult CKD patients [20,24], while controversial effects on endothelial function or arterial stiffness were reported [47]. Significantly lower values of systolic and diastolic blood pressure, consistent with the meta-analysis by Zhang et al. [24], were observed in a retrospective study on a population of PAD patients with concomitant CKD in a high percentage enrolled in the same TiTo program [48].

The lower rate of hospitalizations in the exercise cohort, as well as peripheral revascularizations with a lower aggressive factor for renal function [6,49], also confirmed previous observations. A low rate of interventions compared to the literature was found in PAD patients in a 3-year period after completion of the TiTo program, particularly among those who showed hemodynamic improvements at discharge [50], as well as better clinical outcomes among PAD patients who completed the TiTo program compared to those who interrupted it for non-health reasons [29]. Recently, a lower rate of long-term hospitalization was observed at discharge of a program with the same characteristics in ESRD patients [31,32].

Finally, the differences observed especially in the long-term outcomes may be due to the active lifestyle adopted by patients in the exercise group, who may have performed a sort of maintenance exercise even after the 6-month enrollment period. We have no data in relation to this issue, but the promotion of an active lifestyle in this population is warranted and recommended by international guidelines [1,17].

The study had several limitations. First, this was a retrospective study with all the known limitations of such a design. In particular, a cause–effect relationship between intervention and outcomes cannot be claimed, considering several potential factors that may have affected the results, such as the frailest subject exclusion from the rehabilitation program. However when clinical trials are not available, only retrospective studies enable the comparison of different treatments and to assess the feasibility of prospective studies. We cannot a priori exclude a selection bias of participation, since we cannot know if patients in the control group refused participation in the exercise program or simply were not interested in it, but the two groups were matched as baseline.

The number of PAD-CKD subjects was low in absolute, and this could lead to a possible type II error due to a small sample size and to a possible risk of confounding bias, even though the number of patients enrolled was higher than in studies dealing with CKD patients recently reported in a meta-analysis [24]. We also acknowledge the lack of some clinical data from the control group, which were not available from the database of the department that had followed-up patients, including ABI at discharge and the amount of physical activity performed. Medications changed during the follow-up, even if the same experienced nephrologists visited the whole population, and therefore the possible modifications in medications (e.g., interruption of oral anti-glycemic drugs), may have occurred without any differences in both groups. Finally, the eGFR value was estimated based on creatinine; however, agreement with cystatin-C estimation was reported.

Despite all these significant limitations, considering that renal protection in PAD is an under-addressed aspect and that the present study accounted for a difference of over 800 dialysis sessions in favor of the exercise group, the observation was reported. The aim is to stimulate the planning of further studies on the progression of CKD to ESRD, a desired sanitary, social, pharmacoeconomic, and environmental outcome to be pursued in any way in relation to population aging.

## 5. Conclusions

In conclusion, patients with PAD and concomitant CKD completing a 6-month home-based exercise program showed better long-term stability of renal function and more favorable clinical outcomes than a cohort of PAD patients completing usual care observed in the same timeframe. Whether the protection resulting from the exercise was confirmed in randomized trials, the importance of exercise interventions in PAD-CKD patients would be highlighted.

## Figures and Tables

**Table 1:** Baseline comparison of the two groups under study.

	**Exercise** **(n = 32)**	**Control** **(n = 34)**	***p***
Age, years	72 ± 10	72 ± 9	0.82
Male sex, n(%)	27 (80)	25 (78)	0.90
Risk factors and comorbidities			
Smokers	22 (68)	21 (62)	0.54
Hypertension	27 (84)	32 (94)	0.18
Hyperlipidemia	13 (40)	14 (40)	0.78
Diabetes	19 (59)	17 (50)	0.44
Myocardial infarction	16 (50)	17 (50)	1.00
Coronary revascularization	17 (53)	17 (50)	0.89
Heart failure	4 (13)	6 (18)	0.54
Chronic obstructive pulmonary disease	5 (16)	3 (9)	0.47
Neoplastic disease	7 (22)	6 (18)	0.77
Charlson Comorbidity Index	6 ± 2	6 ± 2	0.87
Primary nephropathy, n (%)			
Nephroangiosclerosis	9 (28)	16 (46)	0.17
Diabetic nephropathy	18 (56)	14 (42)	
Single kidney condition	5 (16)	2 (6)	
Others	0 (0)	2 (6)	
Medications, n (%)			
ACE-inhibitors	4 (12)	3 (9)	0.63
Diuretics	23 (72)	28 (82)	0.32
Beta-blockers	14 (44)	20 (58)	0.22
Calcium antagonists	14 (44)	7 (21)	0.10
Statins	14 (44)	16 (47)	0.79
Antiplatelets	20 (62)	24 (71)	0.31
Anticoagulants	14 (44)	9 (26)	0.14
Oral antidiabetic drugs	1 (3)	1 (3)	0.97
Insulin	15 (47)	14 (41)	0.55
Laboratory parameters			
Hemoglobin, g/dL	11.8 ± 1.9	11.7 ± 1.4	0.98
Albumin (%)	52.0 ± 6.0	54.0 ± 5.5	0.54
Serum creatinine, mg/dL	2.35 ± 1.2	2.30 ± 1.0	0.69
eGFR, 6 mL/min/1.73 m^2^	29 ± 2	30 ± 2	0.66
Peripheral artery disease			
ABI more impaired limb	0.64 ± 0.14	0.63 ± 0.17	0.87
Rutherford’s category 2	5 (16)	6 (18)	0.76
Rutherford’s category 3	18 (56)	20 (59)	
Rutherford’s category 4	9 (28)	8 (24)	
Previous lower limbs revascularizations	6 (19)	8 (24)	0.56

eGFR, estimated glomerular filtration rate; ABI, ankle-brachial index.

**Table 2:** Variations in laboratory parameters over time in both groups.

**Ex Group**	**Baseline**	**Year 1**	**Year 2**	**Year 3**	**Year 4**	**Year 5**
S. creatinine (mg/dL)	2.35 ± 0.32	2.43 ± 0.33	2.51 ± 0.33	2.72 ± 0.36	2.79 ± 0.39 *	2.72 ± 0.39 *
eGFR (mL/min/1.73 m^2^)	29 ± 2	29 ± 2	27 ± 2	26 ± 2 *	26 ± 3 *	26 ± 3 *
Hemoglobin (g/dL)	11.8 ± 1.9	11.6 ± 1.6	11.8 ± 1.7	11.6 ± 1.5	12.0 ± 1.5	11.6 ± 1.6
Red blood cells (106/μL)	4.00 ± 0.65	4.25 ± 0.57	4.06 ± 0.43	3.93 ± 0.43	4.06 ± 0.56	3.87 ± 0.59
Hematocrit (%)	36 ± 5	38 ± 5	38 ± 4	35 ± 4	37 ± 4	36 ± 4
Urea (mg/dL)	88 ± 34	89 ± 33	100 ± 60 *	114 ± 58 *	114 ± 53 *	106 ± 40 *
**CO Group**	**Baseline**	**Year 1**	**Year 2**	**Year 3**	**Year 4**	**Year 5**
Serum creatinine (mg/dL)	2.30 ± 0.31	2.86 ± 0.32 *	3.37 ± 0.32 *	3.35 ± 0.33 *	3.25 ± 0.36 *	4.22 ± 0.42 *
eGFR (mL/min/1.73 m^2^)	30 ± 2	25 ± 2 *	23 ± 2 *	24 ± 2 *	23 ± 2 *	18 ± 3 *
Hemoglobin (g/dL)	11.7 ± 1.4	11.6 ± 1.7	11.3 ± 1.5	11.4 ± 1.6	11.8 ± 1.5	11.3 ± 1.5
Red blood cells (106/μL)	4.01 ± 0.59	4.11 ± 0.71	3.91 ± 0.63	4.02 ± 0.60	4.05 ± 0.63	3.80 ± 0.53
Hematocrit (%)	36 ± 5	37 ± 6	35 ± 4	36 ± 4	37 ± 4	36 ± 7
Urea (mg/dL)	86 ± 38	112 ± 53	112 ± 66 *	117 ± 49 *	114 ± 44 *	136 ± 71 *

* *p* < 0.05 with respect to baseline.

## Data Availability

The datasets generated and analyzed during the current study are available from the corresponding author on reasonable request. Data is not publicly available due to privacy or ethical restrictions.
